# Spheroid Size Does not Impact Metabolism of the β-blocker Propranolol in 3D Intestinal Fish Model

**DOI:** 10.3389/fphar.2018.00947

**Published:** 2018-08-22

**Authors:** Laura M. Langan, Stewart F. Owen, Maciej Trznadel, Nicholas J. F. Dodd, Simon K. Jackson, Wendy M. Purcell, Awadhesh N. Jha

**Affiliations:** ^1^School of Biological and Marine Sciences, University of Plymouth, Plymouth, United Kingdom; ^2^Global Sustainability, AstraZeneca, Macclesfield, United Kingdom; ^3^College of Life and Environmental Sciences, University of Exeter, Exeter, United Kingdom; ^4^School of Biomedical and Healthcare Sciences, University of Plymouth, Plymouth, United Kingdom; ^5^Harvard T.H. Chan School of Public Health, Harvard University, Cambridge, MA, United States

**Keywords:** spheroids, EPR-oximetry, micro-environment, metabolism, intestine, β-blocker, propranolol

## Abstract

Compared to two-dimensional (2D) cell culture, cellular aggregates or spheroids (3D) offer a more appropriate alternative *in vitro* system where individual cell-cell communication and micro-environment more closely represent the *in vivo* organ; yet we understand little of the physiological conditions at this scale. The relationship between spheroid size and oxygen microenvironment, an important factor influencing the metabolic capacity of cells, was first established using the fish intestine derived RTgutGC cell line. Subsequently, pharmaceutical metabolism (Propranolol), as determined by high performance liquid chromatography, in this intestinal model was examined as a function of spheroid size. Co-efficient of variation between spheroid size was below 12% using the gyratory platform method, with the least variation observed in the highest cell seeding density. The viable, high oxygen micro-environment of the outer rim of the spheroid, as determined by electron paramagnetic resonance (EPR) oximetry, decreased over time, and the hypoxic zone increased as a function of spheroid size. Despite a trend of higher metabolism in smaller spheroids, the formation of micro-environments (quiescent, hypoxic or anoxic) did not significantly affect metabolism or function of an environmentally relevant pharmaceutical in this spheroid model.

## Introduction

The spheroid (three-dimensional or 3D) culture system is a model used increasingly in study function, metabolism and toxicological assessment of drugs and contaminants across a broad range of biological systems (Sutherland et al., 1981; Ma et al., 2003; Xu et al., 2003; Curcio et al., 2007; Hirschhaeuser et al., 2010; Baron et al., 2012; Mehta et al., 2012; Cueto et al., 2013; Uchea et al., 2015). While microenvironment gradient formation has been well characterized in terms of oxygen diffusion in tumor based spheroid models (Mueller-Klieser, 1984; Sutherland et al., 1986; Vaupel et al., 1989; Hashem et al., 2015), questions still remain in terms of nutrient and xenobiotic diffusion/or metabolism in non-tumorous spheroid models. Beginning with the route of administration and uptake to the final site of action, a drug/toxicant encounters numerous barriers and transporters before reaching its target. Transporters play a key role in the uptake of toxicants and drugs by cells, with the largest family of transporters known as the ATP binding cassette (ABC) which include *P*-glycoprotein (Pgp). These proteins function as active transporters for multiple substrates across the cellular membrane and are highly conserved across animal taxa (Jeong et al., 2017). However, in cells cultivated as a 3D construct, oxygen is transported by diffusion and simultaneously consumed by cells (Curcio et al., 2007). In this context, a clear trend emerges where larger spheroids demonstrated markedly distinct diffusional gradients which do not provide sufficient nutrients into the core of the aggregate. As a consequence of limited nutrients, basic cellular activity such as waste removal is reduced resulting in eventual cell death. Any adjustment to this diffusion has clear impacts on energy and xenobiotic metabolism and subsequently can impact overall physiology and function of the spheroid. Typically, studies which address energy metabolism limitations in these models have used mathematical modeling of growth (Venkatasubramanian et al., 2006; Curcio et al., 2007) or cytotoxic changes in terms of quantitative changes in glucose, LDH or protein (Ma et al., 2003; Xu et al., 2003) as a means of minimizing the degree of variation between predictions and reality. Regardless of the obvious connection, few studies address the differences in xenobiotic metabolism as impacted by spheroid size. Cytotoxic response to numerous pharmaceutical compounds using spheroids derived from both a primary source and a cell line was compared (Xu et al., 2003), but no attempt was made to correlate metabolic rate with respect to spheroid cell seeding density, size and growth.

Significant differences in responsiveness of different 3D models following exposure to chemicals are difficult to interpret since there is no agreed or standardized spheroid size, or cell seeding protocol. Substantial advances in our knowledge about these 3D models have highlighted the potential impact of spheroid size in terms of their metabolic capability and responsiveness. For example, recent studies identified a maximum spheroid size where cells are exposed to sufficient oxygen in order to prevent or minimize hypoxia in their internal structure but did not quantify the metabolic differences between size categories (Curcio et al., 2007; Langan et al., 2016). It is unsurprising that reviews have focused on the incorporation of spheroid size in terms of responsiveness to treatment (Hirschhaeuser et al., 2010) and on opportunities and challenges associated with the use of this model to test drug delivery and efficacy (Mehta et al., 2012). However, development of high-throughput preparatory methodologies rarely addresses the impact of spheroid size and generally, the focus is drug penetration within a spheroid in relation to the molecular structure of the drug. Given that metabolic capacity of spheroids could depend on the size of spheroids, one might argue that the impact of size is important. If different size categories of spheroids are known to have variable zones of proliferating cells, necrosis or oxygen transport limitations, how can a single response be standardized or compared *in vivo* or *in vitro* in any animal or human system? Obviously, this has significant implications while extrapolating the information for fundamental biological or clinical understandings.

Previously we have addressed one of the fundamental unknowns for the widespread use of spheroid models by elucidating oxygen micro-environment in spheroids in fish cell line (i.e., RTG-2) derived spheroids of various sizes (Langan et al., 2016). With precise cultivation of the spheroid size in terms of cell seeding density, zones of quiescence can be kept to a minimum and consequently unmodified metabolism can occur within the model. Yet, questions regarding fundamental characterization of how cell seeding density/spheroid size affects the oxygen micro-environment and the xenobiotic metabolism of the model system are still not available in the literature. Despite an acknowledged need, there is a lack of established cell lines derived from the gastrointestinal tract of fish to help understand dietary exposure to chemicals in the aquatic environment (Langan et al., 2017). The use of the intestinal RTgutGC cell line to model metabolism in the form of a cellular aggregate therefore provides the opportunity to elucidate the metabolism of environmentally relevant contaminants in the gut environment in a more complex culture system. As described in later sections, this cell line cultured as spheroids demonstrates comparative morphology to the native intestine in the form of numerous polarized micro-villi formations on the edge of the spheroid structures which facilitates metabolism in this system and has been previously observed in the cell line when cultured on Transwell^®^ inserts (Langan et al., 2017). Staining for the presence of mucosubstances revealed strong staining for both acidic and neutral mucosubstances indicative of goblet cells which are comparable to expression of the intestine *in vivo*. Furthermore, available information also suggests an increasing use of intestinal spheroid cultures (McCracken et al., 2011; Mustata et al., 2013; Schlaermann et al., 2014), with increasing demand for teleost derived spheroids as the requirement for animal alternatives for fish ecotoxicological studies increases (Lillicrap et al., 2016).

Previous studies from our laboratories have demonstrated that the β-blocker propranolol, an environmentally relevant pharmaceutical, is actively metabolized both by hepatocytes and in spheroids derived from rainbow trout liver cells (Bartram et al., 2012; Baron et al., 2017). Despite the importance, as mentioned above, *in vivo* dietary uptake studies for chemicals in general and pharmaceuticals such as propranolol are not well reported in fish or aquatic models. Uptake of propranolol in the intestine has been reported to be less than half that of the liver in rabbit (Du Souich et al., 1995) and in murine systems (Okabe et al., 2004; Masaki et al., 2006). Using the intestinal RTgutGC cell line cultured as spheroids and propranolol as our environmentally relevant test compound, we compared and contrasted different size classes of spheroids to test the hypothesis that the size of spheroids impact xenobiotic metabolism. To test this hypothesis, we used electron paramagnetic resonance (EPR) oximetry to determine oxygen gradients within this system as a function of spheroid size. We further characterized the functionality of the cell line using histochemical staining. To complement the determination of oxygen levels in different spheroid sizes, we used high performance liquid chromatography (HPLC) to determine the active metabolism of propranolol.

## Materials and Methods

### Reagent and Stock Preparation

All reagents were from Sigma-Aldrich (United Kingdom) unless otherwise indicated. Propranolol hydrochloride (99% pure, CAS 318-98-9, C_16_H_22_O_2_NCl) was supplied by industrial partner (AstraZeneca). Preparation of the oximetry probe LiPc (Lithium phthalocyanine) was carried out as previously described in detail (Langan et al., 2016).

### Cell Culture

The rainbow trout gastrointestinal cell line RTgutGC was a kind gift from Dr. Lucy Lee (University of Fraser Valley, Canada) (Kawano et al., 2011). The cell line was routinely cultured in our laboratory using 75 cm^2^ culture flasks at room temperature (21°C) in L-15 culture medium supplemented with 10% FBS (Langan et al., 2017). Cells were seeded at a density of 5 × 10^4^ cells/mL, and became confluent 7–8 days later. Spheroids were formed as previously outlined for another fish cell line (Langan et al., 2016). Subsequent experiments were carried out on spheroids of three initial seeding densities (i.e., 12.5, 50 and 100 × 10^4^ cells/mL), with a volume of 200 μL per spheroid well.

### Morphological Characterization of the RTgutGC Spheroids

The growth of individual spheroids was calculated according to the methodology established previously in our laboratory (Baron et al., 2012). Morphological characterization of the RTgutGC spheroid was observed under light microscopy over 14 days, with surface structure observed under Transmission Electron Microscopy (TEM) and histological staining of mucosubstances following previously established protocols (Langan et al., 2017).

### Electron Paramagnetic Resonance (EPR) Oximetry

Oxygen gradients across the spheroids were quantified as previously described by us (Langan et al., 2016). Briefly, spectra were recorded on a Bruker EMX micro EPR spectrometer fitted with variable temperature accessory, operating at 9.4 GHz. Spheroids (2 per spectra recording) were drawn into the PTFE gas permeable tubing with ∼5 μL of medium per spheroid. The tube was folded once, and the spheroids allowed to sediment at the fold. Samples were maintained in the cavity at 292°K, which is equivalent to the incubation temperature (19°C). Oxygen saturation was quantified by measuring the peak to peak line width of the spectrum relative to a sample which contained only the probe and medium but not spheroids/cells. As per previous experiments (Langan et al., 2016), the zone of hypoxic and anoxic cells was quantified based on mathematical calculations (Grimes et al., 2014) following the identification of viable rim from EPR recordings.

### Preparation of Spheroids for Exposure

Spheroids (7 days old) were pooled (for each separate experiment) from 96-well micro plates into a clean 96-well pHEMA-coated microplate at a seeding density of 12 spheroids per well of the 5 × 10^4^ cells/mL seeding and 6 spheroids per well of the 10 × 10^4^ cells/mL cell seeding density (in 75 μL L-15 medium; pH 7.4). These spheroid sizes were chosen as they adequately represent the range of spheroid radii typically reported in the non-tumor spheroid literature. For exposure, the spheroids were exposed to 75 μL of a solution of 200 μg/L of propranolol (final concentration 100 μg/L in well) reconstituted in L-15 medium and incubated at 15°C for 24 h. Control samples were quenched immediately after the addition of the exposure solution to samples using acetonitrile.

### Metabolism of Propranolol in the Spheroids

Analyses were performed using Surveyor MS Pump Plus HPLC pump with HTC PAL autosampler coupled to TSQ Vantage triple quadrupole mass spectrometer equipped with heated electrospray (HESI II) source (all ThermoFisher Scientific, Hemel Hempstead, United Kingdom). Chromatographic separation was achieved using reversed-phase, 3 μm particle size, C18 Hypersil GOLD column 50 mm × 2.1 mm i.d. (Thermo Scientific, San Jose, CA, United States). Analytes were separated using a linear gradient of aqueous phase and Methanol both containing 0.1% of Formic Acid. Methanol content increased from 20% to 100% in 1.5 min and maintained for another 1.5 min before returning to the initial condition. The flow rate was 500 μL/min. Temperature of autosampler was set at 8°C while column was kept at a room temperature. HESI probe was operating in positive mode while an ion-spray voltage of +3.75 kV was applied. The heated capillary temperature was set at 270°C and the vaporizer temperature was 350°C. Nitrogen was employed as sheath and auxiliary gas at a pressure of 60 and 2 arbitrary units, respectively. The argon CID gas was used at a pressure of 1.5 mTorr. Quantification of propranolol was performed by monitoring characteristic multiple reaction monitoring (MRM) transitions 260.2→116.2 m/z (collision energy 17 eV). Internal standard method of quantification, using matrix matched standards was used in all samples analysis.

Additionally, CYP3A quantification in the intestinal spheroid model using the model inducer Testosterone was also examined (Kamiguchi et al., 2010). CYP3A activity was quantified by measuring the extent of 6β-hydroxytestosterone (H2898, CAS: 62-99-7; Sigma) formation from testosterone using ultraperformance liquid chromatography systems (UltiMate 3000, ThermoScientific). Stock solutions were made up in HPLC grade methanol (Sigma, United Kingdom). Briefly, the cultured RTgutGC cells were seeded at varying densities and washing thrice with Hanks balanced salt solution and then incubated with 100 μM testosterone (T1500, CAS: 58-22-0; Sigma) for 2 h. At the end of incubation, 250 μL of the media was transferred into a glass vial with 250 μL of methanol and the 20 μL of the mixture injected onto the column. Samples, testosterone and 6β-hydroxytestosterone were chromatographed using Aquasil C18 column (150 mm × 4.6 mm, 3 μm; ThermoFisher) with a C18 security guard (Phenomenex, United Kingdom). The mobile phase consisted of diluted methanol in water with a gradient profile starting at 50% from 0 to 15 min, increasing to 60% until 20 min, increasing again to 85% until 22 min before decreasing back to 50%, all at a flow rate of 1 mL/min). The detection limits for testosterone and 6β-hydroxytestosterone metabolites was 0.1175 μM.

### Statistical Analysis

Statistical analyses were preformed using R, Version 3.1.3 (R Core Development Team, 2015). Data is given as mean values ± SEM, with *n* denoting the number of replicates per experiment unless otherwise indicated. These replicates are representative of non-parallel passages of the cell line, with each recording representative of 3-4 technical replicates. Data were first tested for normality and homogeneity of variance using the Anderson-Darling Normality test and Levene’s test respectively. Analysis of variance (ANOVA) was performed for multiple factor comparisons when assumptions were met. Due to non-normality and variable variance in micro-environment recordings, data was analyzed using a Friedman non-parametric test. A value of *p* < 0.05 was considered significant, with data presented as *p* < 0.001 demonstrating highly significant results.

## Results

### Morphological Characterization

**FIGURE 1:**
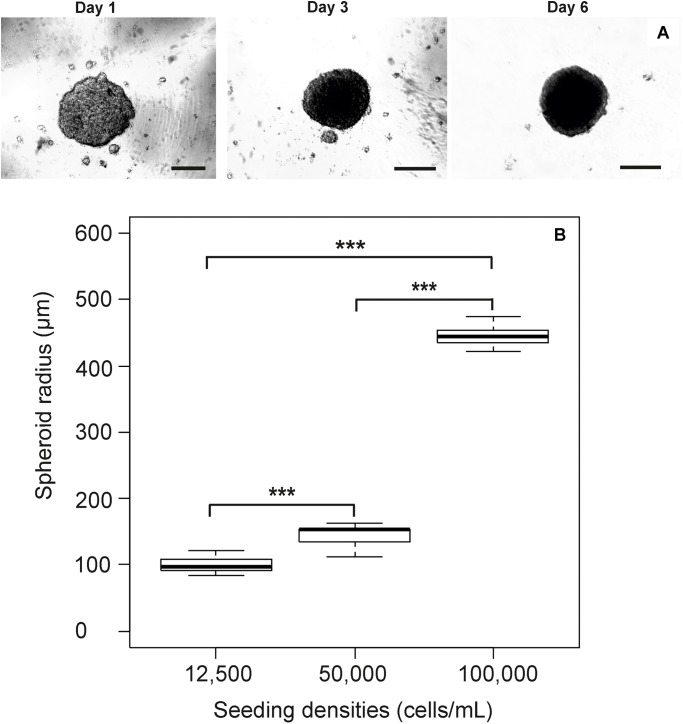
Growth of the RTgutGC cell line as spheroids. Spheroid growth progresses as normal with an obvious incorporation of individual spheroids from day 1 to day 6 (scale bar = 100 μm) where only one spheroid is visible **(A)**, with the impact of modifying initial seeding density during spheroid formation presented **(B)**. A clear trend towards larger spheroids with higher initial cell seeding is clearly visible. Analysis of spheroid size via ANOVA reveals significant difference between all size categories (*p <* 0.001).

**FIGURE 2:**
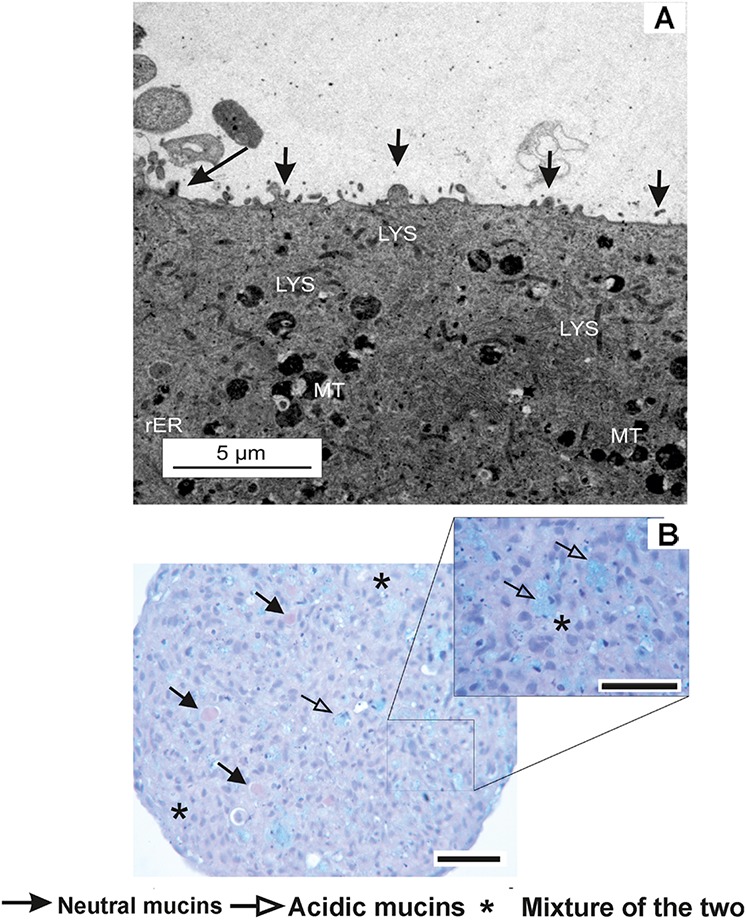
Characterization of the RTgutGC cell line. **(A)** Transmission electron microscopy image of RTgutGC spheroids on day 7 of growth at the lowest seeding density (i.e., 12,500 cells/mL or 2,500 cells/spheroid). Apical protrusions of micro-villus structures are labeled and denoted by arrows. In addition, other common cellular features are visible including rough endoplasmic reticulum (rER), mitochondria (MT) and lysosomes (LYS). **(B)** Histological sections of RTgutGC spheroids generated through gyratory culture. Positive staining for both acidic and neutral mucosubstances via PAS and Schiff stain of a section of spheroid (50,000 cells/mL) on day 7. Presence of both acidic, neutral and mixtures of mucins are comparable to native rainbow trout intestine (Khojasteh et al., 2009). Scale bar = 100 μm.

Prior to the identification of micro-environment formation in the RTgutGC spheroid, it was critical that spheroid growth (across all cell seeding densities) was first established in order to identify the point at which spheroids were formed visually (**Figure 1a**) and to what size spheroids varied dependent on initial seeding (**Figure 1b**). This time point (day 7) was then used as the basis for assessment of micro-environment formation and later for the assessment of metabolism. During formation, the RTgutGC spheroid became significantly more compact, with a reduction in size of 30 – 40% observed irrespective of original seeding density. Transmission electron microscopy revealed microvilli (**Figure 2A**). Mucosubstances in the intestine are known to play an important role in the protection and uptake of xenobiotics from the ingested chyme, and their presence in the RTgutGC spheroid model was confirmed using histological staining (**Figure 2B**). Indeed, the intensity of staining is directly comparable to *ex vivo* adherent cultures of trout intestine (personal observation) and to cultures of the cell line grown on Transwell^®^ inserts (Langan et al., 2017).

### Oxygen Micro-Environment Formation

**FIGURE 3:**
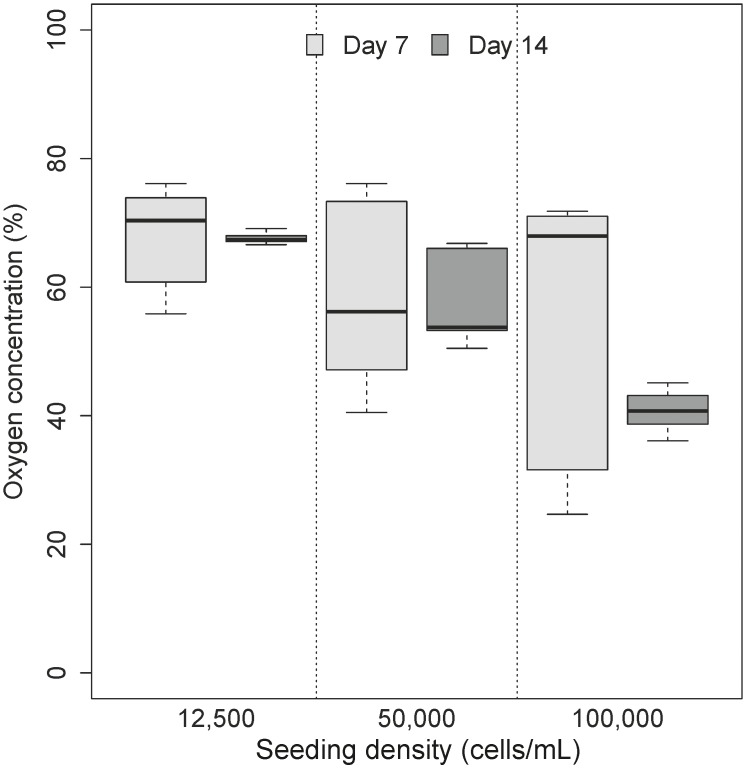
Impact of varying cell seeding densities on the formation of oxygen micro-environments. No significant differences were observed in the formation of oxygen microenvironment (*p >* 0.05). As denoted by the legend, 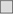 is representative of samples collected on day 7 where cells have become confluent in cell culture model and 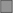 is representative of samples collected on day 14.

Three experiments from non-parallel passages were recorded on day 7 and day 14 to elucidate variation in oxygen micro-environment formation as a function of seeding density. Diameters of the spheroids ranged from 143 to 444 μm (**Table 1**). Coefficient of variation between spheroids was recorded to denote comparability between experiments (and different passages) with an obvious trend toward minimization of variability in the larger size class (**Table 1**). Application of Friedman’s test revealed no significant changes in distribution of micro-environment formation over time or seeding density (χ^2^= 3, *df* = 2, *p* = 0.22) (**Figure 3**).

**Table 1:** Characteristics of the spheroids from the RTgutGC cell line with respect to variation in size as a function of initial cell seeding volume and the formation of micro-environments.

Seeding (Cells/Spheroid)	Sampling (Day)	Passage No. *N* =	Spectra No. *N* =	Δ 0_2_ (%)	CV (%)	Diameter (μm)	Viable Rim (μm)	Hypoxic Zone (μm)
2,500	7	3	14	68 ± 1.5	12	100 ± 5	68	13
	14		14	68 ± 0.2			68	13
10,000	7	3	13	51 ± 2.7	11	144 ± 4	73	41
	14		10	60 ± 2.3			86	29
20,000	7	4	10	54 ± 5.8	3	445 ± 3	269	113
	14		10	41 ± 1.1			181	177


*Data is presented as mean ± standard error of the mean. Data is representative of 3–4 passages with total spectra recordings (denoted by spectra) dependent on spheroid size and time. Clear spectra did not require extra sampling. This is due to 2–3 spheroids on average being used for the smaller size category to obtain a clear line width spectra, while 1–2 spheroids are required for the large size category (20,000 cells/spheroids). Seeding is representative of cells per spheroid (200 μL volume).*

Oxygen saturation was calculated as a percentage of narrowing line width in paramagnetic oximetry (Langan et al., 2016). While all oxygen profiles (in all seeding densities) were characterized by a general decrease in oxygen levels over seeding densities and with time (**Figure 3**), the lack of significant differences between models was not unexpected. Of particular interest is the apparent increase in oxygen concentrations in spheroids with over 10,000 cells seeded which recorded an apparent increase in oxygen with time. Partitioning of the micro-environment within the spheroid model is displayed in **Table 1**. Quiescent zones (where cells are inactive) were determined for each size class investigated (based on differences in viable rim and oxygen diffusion distance), where a consistent trends of zones of 19–20% of total spheroid size were identified for all size classes.

### Metabolism of Propranolol in the Spheroids

**FIGURE 4:**
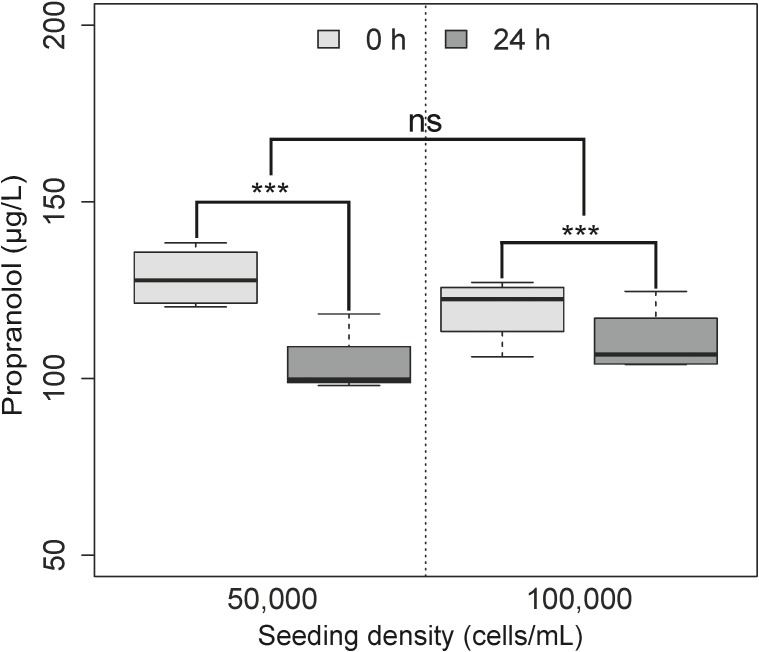
Substrate depletion of propranolol over time using two different cell seeding densities. Each treatment is repeated in quadruplicate over 4 non-parallel passages (*n* = 4). No significant difference in terms of metabolism was observed between the two seeding densities (*p* = 0.80; ns), but was found to be different over time (*p <* 0.001). As previously outlined in **Figure 3**, 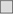 is representative of samples collected immediately following exposure with no active metabolism of product, 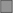 while is indicative of samples collected after 24 h of metabolism by the spheroid model.

All spheroid samples which underwent propranolol exposure were within the analytical limit of quantification, with exposure occurring on day 7 in order to investigate the relationship between metabolism and micro-environment formation. The depletion of propranolol was measured in four non-parallel individual passages (biological variability) of the RTgutGC cell line cultured as spheroids over 24 h, with each treatment in quadruplicate. A Shapiro–Wilk test revealed normal data (*W* = 0.9343, *p* = 0.29), while a Levene test revealed normal homogeneity of variance (*df* = 3, *f* = 0.064, *p* = 0.98), and so an ANOVA was applied to the data. This revealed significant differences between time (*p <* 0.001), but not between seeding densities (*p* = 0.81). Despite the non-significant differences between the two seeding densities, the degree of substrate depletion was different (**Figure 4**) between the lower seeding category of 50 × 10^4^ cells/mL (*n* = 4, CV = 9.23%, 25% difference between 0 and 24 h) and the higher seeding category of 100 × 10^4^ cells/mL (*n* = 4, CV = 2.38%, 15.25% difference between 0 and 24 h).

To investigate if the metabolism of propranolol occurred via the CYP3A pathway, standard curves were generated for testosterone and 6β-hydroxytestosterone as controls for measurement of CYP3A activity in the RTgutGC cell line (2D and 3D). No response was observed in cells seeded at any of the concentrations used, although numerous methodologies were tried including increasing seeding densities, incubation time, or indeed analytical chemistry by increasing injection volume from 20 to 100 μL, which caused no significant improvement in resulting peak (data not shown). In order to ascertain whether testosterone as a CYP3A inducer is cell line specific through a minimization of enzyme activity in cell line versus native tissue (via de-differentiation), primary cultures of rainbow trout intestine were used as controls, with cells exposed directly rather than allowed to attach to substrate (a suspension culture). Primary culture observations indicate that trout intestine does not directly metabolize testosterone to 6β-hydroxytestosterone as predicted in other tissue models (e.g., liver) (Nabb et al., 2006).

## Discussion

Propranolol metabolism is complex. In mammals we understand there are three hepatic pathways: hydroxylation of the naphthalene ring, and two processes focused on the decomposition of the side chain, that are glucuronidation and *N*-desisopropylation that together provide 14 potential metabolites (see **Supplementary File** for details and diagrams). In fish we understand little of which of these potential paths and processes are active. In this study, different sized spheroids initiated through augmentation of initial cell seeding were characterized. As previously observed using the RTG-2 cell line, spheroid diameter appears to stabilize after 7 days in culture over all seeding densities investigated (Langan et al., 2016). Until recently, little attention was devoted to the impact that spheroid size will play on active metabolism, but a recent review reiterates the importance of spheroid size with respect of response to treatment in multicellular tumor spheroids (Hirschhaeuser et al., 2010). Of note is the emphasis placed on the establishment of pathophysiological gradients or micro-environments where proliferative cells are arranged in a concentric pattern around a hypoxic/necrotic core, which is common in tumor formation. Unusually, the establishment of this micro-environment was also shown by us in another fish cell line derived spheroids, with an increase in hypoxic zone congruent to increased size (Langan et al., 2016), a finding also observed in the present study with RTgutGC derived spheroids. Remarkably, although similar seeding densities were used in both studies and spheroid size was comparable (e.g., at a seeding of 100 × 10^4^ cells/mL, spheroids of size 445 ± 3 and 450 ± 43 μm were obtained for the RTgutGC and RTG-2 cell lines respectively), oxygen saturation differed markedly (∼20%) between the two models with RTgutGC equivalent to 54% oxygen saturation and RTG-2 equivalent to 34% on day 7. In addition, spheroid size, diffusion distance and quantification of viable rim also differed between these two models, and among different seeding densities and spheroid size. The difference between models highlights the importance of characterizing each model derived from different cell line/tissue origin, including for spheroids originating from different tumors. Assuming broad comparability could provide misleading biological information particularly when assessing metabolism and biological responses of different chemicals or drugs including for spheroids derived from different malignant cell lines which have many clinical applications. This also appears fundamentally important for the application of theoretical or mathematical approaches to characterize zones in spheroid models.

The transport of compounds through cell membranes is a well-known feature in the determination of xenobiotic detoxification. This feature has a key impact on absorption potential, distribution, elimination, toxicity and especially efficacy of compounds as noted in cancer spheroid models (Achilli et al., 2014). Spheroids are aggregates of cells derived from either primary or established cell lines which have been shown to recapitulate some native architecture and capacity for xenobiotic metabolism and elimination of compounds (Hirschhaeuser et al., 2010; Baron et al., 2012; Mehta et al., 2012; Uchea et al., 2015). However, they suffer from some limitations in terms of micro-environment formation (Mehta et al., 2012). Yet this formation of micro-environment can be minimized following thorough characterization and optimization of model particulars (Langan et al., 2016). Importantly, until recently thorough characterization of fish spheroid models in terms of micro-environment formation has been lacking. This could potentially influence wider application of this animal alternative system through valid concerns regarding their capacity to replace complex *in vivo* animal systems. In this study, the use of the fish derived intestinal RTgutGC cell line cultured as a spheroid has previously demonstrated comparative morphological developments in terms of numerous polarized micro villi formations (with glycocalyx coatings) on the edge of the spheroid structure indicative of goblet cells which are comparable to expression of *in vivo* trout intestine. In addition, unlike those spheroid models which increase in size over time, the RTgutGC model appears to condense over time (across all seeding densities) becoming a stable volume at 7 days as previously reported by us in the RTG-2 cell line (Langan et al., 2016). Two explanations for this phenomenon are proposed. Compression of spheroid size over time may be directly related to cells achieving confluence under normal growth conditions, which has been previously reported in other spheroid models (Alessandri et al., 2013). However, an alternative explanation is also proposed surrounding the cellular architecture itself. Examination of the RTgutGC cell line during monolayer growth reveals attachment of the cells using elaborate interdigitations (interlocking of parts by finger-like processes) to connect cells. We postulate that the decrease in spheroid size over time is due to connections between these interdigitations increasing the cellular connectivity. As a consequence, spheroid compactness naturally increases and would result in the increased expression of tight junctional proteins such as ZO-1 and *E*-cadherin.

As demonstrated in the current study, the RTgutGC cell line is capable of metabolizing propranolol (indicated through compound depletion) with metabolic rate being linked with spheroid size. The use of spheroids in uptake and diffusion studies is increasingly revealing new insights pertaining to drug metabolism and effects in the cellular environment (Achilli et al., 2014). However, it is important to note that such studies used smaller sized spheroids (<100 μm) which could have an impact on the rate of metabolism. Our study demonstrates that for the fish based intestinal RTgutGC model, spheroids below the size of 500 μm do not demonstrate a significant impact on xenobiotic metabolism. Yet, examination of the results suggest a trend toward higher metabolism in the smaller spheroid size (143 ± 4 μm; 50 × 10^4^ cells/mL) at 25% than compared to the larger spheroid (445 ± 2.56 μm; 100 × 10^4^ cells/mL) at 15%. This suggests that metabolic activity within this model may be directly related to the viability and activity of the cells within the spheroid, with larger spheroids having demonstratively less oxygen diffusing into the system then their smaller counterparts. As a consequence of this limited amount of oxygen in larger spheroids, cellular stress is prevalent and thereby the scope for normal cellular activity in terms of metabolism is reduced to maintain basic functionality in the larger spheroids. Interestingly, a strong negative relationship was found between metabolism and the formation of zones of hypoxia and quiescence, which would suggest that diffusion from external media to the cellular body is significantly influenced by the formation of boundaries in spheroid models. As would be expected, the spheroid size and cell seeding show significantly strong positive relationships with micro-environment formation, a finding supported by tumor models which note that boundary formation is influenced mainly by oxygen diffusion limits (Grimes et al., 2014).

The liver has long been considered the primary organ responsible for drug metabolism, with several studies addressing hepatic clearance of propranolol in both murine (Xu et al., 2003; Okabe et al., 2004) and aquatic systems (Owen et al., 2009; Bartram et al., 2012; Baron et al., 2017). Owen et al. (2007) has also compared and contrasted hepatic clearance of β-blockers between mammals and fish. However, in fish, Randall et al. (1998) first suggested that uptake of this drug was most likely to occur via the gills due to its low lipophilicity (log *D_ow_*= 1.2, pH 7.2), a finding supported recently by Stott et al. (2015) who identified rapid uptake of propranolol in the gills of rainbow trout. Although a general consensus emerges in the literature of hepatic first pass metabolism for propranolol, recent studies have recognized that the liver is not singularly responsible for all xenobiotic metabolism. Indeed, most drug metabolizing enzymes present in the liver are also found in the intestine albeit at much lower levels. For example, in rabbits, propranolol uptake was observed in the intestine at 43% (liver of 96–97%) (Du Souich et al., 1995), while rat models have also demonstrated active metabolism in the intestine via simple passive diffusion (Masaki et al., 2006). Our results suggest that propranolol uptake in the intestinal cells occurs via transcellular pathways given that spheroids needed to be disaggregated in order to measure propranolol in the experimental samples. While the suggestion of transcellular uptake is supported in the murine intestinal literature (Dixit et al., 2012), data collected during our study of the metabolism of propranolol within this intestinal cell line suggests that these processes are not singularly controlled by the CYP pathways and warrant further investigations. The current study emphasizes that a large suite of chemicals still need to be tested with fish based spheroid systems in order to reach a consensus on whether micro-environment formation in these constructs will negatively impact on the xenobiotic metabolism of compounds with diverse modes and mechanisms of action. In this context, as mentioned earlier, it is crucial to investigate differences between spheroids derived from different tissues (cell lines), primary cultures or laboratory animals. This may have significant impacts in the form of type I or type II errors in hazard assessment if these constructs were adopted in a regulatory context. Although recent research from our laboratory suggested that fresh hepatocytes cultured as spheroids demonstrate some aspects of the *in vivo* situation with respect to compounds such as propranolol and diclofenac, it was suggested that further studies are urgently needed at both *in vitro* and *in vivo* level to determine the sensitivity of 3D models in predicting whole animal responses with respect to biotransformation (Baron et al., 2017).

Studies evaluating and supporting the combined testing of both intestinal and hepatic metabolism, either *in vivo* or *in vitro* are increasing (Triebskorn et al., 2004; Martignoni et al., 2006; Masaki et al., 2006; Kajbaf et al., 2013). Research to improve the complexity of *in vitro* toxicity assessment has seen an increase in the reporting of co-culturing of various organs/cell types to address the biological complexity of uptake (e.g., Abu-Absi et al., 2004; Yoshikawa et al., 2011; Le Hegarat et al., 2012). Intrinsic to this is the inherent difference in metabolic enzymes between organs being a significant driver of observed metabolism difference. Previous studies have supported the use of testosterone as a control CYP3A inducer in both intestinal and hepatic cultures (Watanabe et al., 2007; Kamiguchi et al., 2010), however, this induction was not repeatable using the RTgutGC cell line which produced no discernible metabolites when compared to standards. A suggestion for this lack of metabolite formation is suggested in the mammalian literature where Martignoni et al. (2006) reported higher levels of 6β-hydroxytestosterone in the liver compared to the intestine, and the reverse of this trend for 16-β-hydroxytestosterone. This would suggest the requirement of a different product as a control substance to measure metabolic activity in the intestinal cell line. In rat intestinal microsomes, Kajbaf et al. (2013) previously reported a lack of testosterone transformation to 6-β-hydroxytestosterone, while James et al. (2005) reported that fish diet and intestinal region can also influence CYP3A activity, with little activity reported in the distal region of the intestine (mid and posterior). The current study found no active transformation of testosterone in the RTgutGC cell line which was originally developed from the distal intestine of rainbow trout. However, when fish intestinal primary cultures are contrasted, a different picture emerges which is more comparable to the mammalian *ex vivo* literature whereby three unknown peaks are suggested by the chromatogram, and no difference observed between intestinal regions. The presence of CYP3A and the efflux transported *P*-glycoprotein have already been established in RTgutGC cell line under different exposure conditions (Langan et al., 2017) indicating its metabolic competence, and the lack of metabolism of testosterone does not appear to impact the metabolism of propranolol outlined in this study. Foreseeable reasons for this are found in murine systems which reports that propranolol is taken up via simple diffusion without the participation of efflux transporters at an apical pH of 7.4 (Masaki et al., 2006). However, most previous studies of fish CYP metabolism in pollutant biotransformation have focused on the CYP1A family, with further studies required on the induction of CYP3A (James et al., 2005).

As previously outlined, most studies do not justify the use of small (or large) spheroids for drug uptake to investigate metabolism. The data indicates that spheroids with a size of less than 200 μm would be preferable to use when extreme micro-environments which can affect cellular metabolism should be kept to a minimum. To the our knowledge, the present study presents the first study addressing why the use of different sized spheroids may negatively/adversely impact uptake and xenobiotic metabolism through the formation of micro-environments which could be detrimental to cellular health and activity in a non-tumor spheroid model. In order to utilize the spheroid model, a thorough characterization of all spheroid models derived from different organs should be undertaken to establish baseline data to facilitate comparison. In this instance, we have begun this with the current study and previous work (Langan et al., 2016). Given the minimal information available in terms of dietary uptake of contaminants, it is, however, vital that organs such as the liver are also investigated to compare differences in metabolic response to really understand the benefits and limitations of these 3D constructs.

## Author Contributions

LL, SJ, WP, ND, and AJ conceived and designed the experiments. LL, ND, and MT performed the experiments. LL and MT analyzed the data. LL, SJ, SO, and AJ contributed to the writing of the manuscript. SO, ND, MT, SJ, and AJ critically reviewed and revised the manuscript.

## Conflict of Interest Statement

The authors declare that the research was conducted in the absence of any commercial or financial relationships that could be construed as a potential conflict of interest. The reviewer ORP and handling Editor declared their shared affiliation.
