# Supplementary material for: Spheroid Size Does not Impact Metabolism of the β-blocker Propranolol in 3D Intestinal Fish Model
Source: Front Pharmacol. 2018 Aug 22;9:947. doi: 10.3389/fphar.2018.00947 (PMC6113889; doi:10.3389/fphar.2018.00947)
Supplement: Supplementary file 1 [file Table_1.docx]

**Supplemental file**

**Spheroid size does not impact metabolism of the *β*-blocker propranolol in 3D intestinal fish model**

Laura M. Langan^1^, Stewart F. Owen^2^, Maciej Trznadel^3^, Nicholas J.F Dodd^4^, Simon K. Jackson^4^, Wendy M. Purcell^5^, Awadhesh N. Jha^1^*^,^*^∗^

^1^*University of Plymouth, School of Biological and Marine Sciences Plymouth, UK*

^2^*Global Sustainability, AstraZeneca, Alderley Park, Macclesfield, Cheshire, UK*

^3^*University of Exeter, Biosciences, College of Life and Environmental Sciences, Exeter, UK*

^4^*University of Plymouth, School of Biomedical and Healthcare Sciences, Plymouth, UK*

^5^Harvard T.H. Chan School of Public Health, Harvard University, USA

Correspondence*:

Awadhesh N. Jha

[a.jha@plymouth.ac.uk](mailto:a.jha@plymouth.ac.uk)

**Summary**

In this supplemental information we provide in section 1 a summary review of the metabolism of propranolol in mammals as we understand it from the literature. To date it is unclear of these pathways is utilised in fish and in which species.

In section 2 we provide a comparative chromatogram of propranolol metabolites from a 14C-propranolol *in vitro* suspension of rat and trout liver hepatocytes exposed at 20ºC to 1mmol propranolol for 48h. These are preliminary and illustrative only for the range of labelled peaks in each case. We have labelled the time where we expect glucuronides, and the sulphates to appear. For interest we also provide a table summarising the presence or absence of metabolite peaks recovered for trout and representative mammal models showing both gender and species differences.

# Propranolol metabolism

Propranolol undergoes at least three different processes of decomposition in the body resulting in 14 known metabolites (Table 1), of which around 90% of a propranolol dose is recovered as metabolites in the urine (in normal man: Walle *et al*., 1985). The biochemical processes can be divided into the hydroxylation of the naphthalene ring (27-59% of the metabolism), and two processes focused on the decomposition of the side chain, that is glucuronidation (10-25% of the metabolism) and *N*-desisopropylation (32-50% of the metabolism) (Walle *et al*., 1985) [Figure 1]. Of the 14 metabolites, 70% of the propranolol dose may be explained by only four major metabolites (Sowinski *et al*., 1996). These are propranolol glucuronide (PG), naphthoxyacetic acid NLA, 4-OH propranolol sulphate (HOPS), 4-OH propranolol glucuronide (HOPG). The proportion of each process that contributes to each of the three methods of metabolism is very much dependant upon the sex, physiological state and genetic polymorphism of the individual. However, a generalisation of the metabolite production in normal man (Walle *et al*., 1985) is used as a guide to the contribution to each pathway to the metabolite production.

**Figure 1**. First pass pathways of propranolol metabolism in mammalian hepatocytes.

## **Hydroxylation of the naphthalene ring (27-59% of the metabolism).**

Ring oxidation is the primary metabolic pathway for the metabolism of propranolol. It occurs in hepatic microsomes and is mediated by cytochrome P450 (CYP2D6 in humans and CYP2D1 in rats (Masubuchi *et al*., 1994; Walle *et al*.,1996)). Isomer selection is known to vary among species in the degree of isomeric selection that actually takes place (Narimatsu *et al*., 2000). The degree of isomeric selection of propranolol by these P450s is unpublished for fish. Hasler and workers (1999) also report involvement of cytochrome P450 (CYP2C19) in the metabolism of propranolol, although the precise stage that this enzyme takes effect is unclear from the literature.

The propranolol ring is first oxidised to form 4-OH propranolol (HOP) and then to either 4-OH propranolol sulphate (HOPS) or 4-OH propranolol glucuronide (HOPG) (Sowinski et al., 1996). Additionally 5-OH propranolol and 7-OH

propranolol are also reported to be produced in small amounts in the primary ring oxidation, although whether each can go on to produce a sulphate or glucuronide is not evident from the literature but could potentially provide an additional six metabolites, but in reality this may not happen.

## **Side chain *N*-desisopropylation (32-50% of the metabolism).**

First pass side chain metabolism in the liver of mammals is illustrated in **figure 1**. In humans at least, propranolol undergoes enzymatic desisopropylation by a cytochrome P450 (CYP1A2) that is actually an isomer selective hydroxylation to form *N*-desisopropylpropranolol (NDP). Wu and workers (2001) found that the major metabolite from NDP metabolism (accounting for more than 50% of the propranolol) was propranol glycol (PGL) but was very much pH dependant. This was produced via oxidative deamination of NDP within the hepatic mitochondria via an enzymic pathway using monoamine oxidase (MAO) to form an Aldehyde Intermediate (AI). The AI is then reduced by Aldehyde Reductase to form the PGL. NDP is also conjugated to an acetyl NDP, but this metabolite was found to be a minor route. A further unknown route of NDP metabolism accounts for a small proproprtion (<10%) of the metabolic processing of NDP. The AI was also found to give naphthoxylactic acid (NLA) via mitochondrial Aldehyde dehydrogenase (ALDH) and on to naphthoxyacetic acid (NAA) as minor metabolites. It is suggested that the formation of the Aldehyde leads to the toxic effects of propranolol since it can form Schiff bases (bond between the carbonyl group of the ketose substrate and the amino group) with proteins and interact with thiol groups (Wu *et al*., 2001). It has also been noted that polymorphism in the ALDH of humans can result in different responses and alter the toxic response to propranolol such as is seen in the difference between European and Asian human populations (Imamura *et al*., 2002) and other polymorphic variations in the cytochrome P450 enzymes that result in changes in drug pharmokinetics (Hasler *et al*., 1999). Propranolol clearance is also faster in men than women due to faster side chain oxidation associated with higher testosterone levels (Walle *et al*., 1996).

**Figure 2.** First pass, side-chain metabolic pathway of propranolol as determined in rat hepatocytes. The figure represents information combined from Wu *et al*., 2001 and Imamura *et al*., 2002.

## **Side chain glucuronidation (10-25% of the metabolism).**

Glucuronidation is the substitution of the side chain hydroxide for glucose to form the metabolite propranolol glucuronide.

**Table 1.** List of 14 mammalian hepatic metabolites of propranolol that can be collected from urine.

| 70% of metabolites: | 30% of metabolites: |
| --- | --- |
| propranolol glucuronide (PG),  naphthoxyacetic acid (NLA),  4-OH propranolol sulphate (HOPS),  4-OH propranolol glucuronide (HOPG). | *N*-desisopropylpropranolol (NDP).  propranol glycol (PGL),  acetyl *N*-desisopropylpropranolol (AcNDP)  naphthoxylactic acid (NLA)  naphthoxyacetic acid (NAA)  4-OH propranolol (HOP)  5-OH propranolol  7-OH propranolol  Unidentified NDP derivative^1^  Unknown^2^ |

^1^ Unidentified product of NDP as part of the mass balance of Wu et al., (2001).

^2^ Un-described 14^th^ metabolite not apparent from the literature but included by Walle *et al*., (1985) in a total of 14 phase I metabolites of propranolol.

# Propranolol metabolism in livers

**Figure 3.** Two illustrative HP-LC chromatograms demonstrating 14C labelled metabolites of propranolol in rat and trout liver suspensions. Exposure to 1mmol propranolol parent was for 48h at 20ºC in both cultures.

**Table 2.** Table illustrating presence or absence of metabolites in a range of mammals and trout. This is only intended to point towards the complexity in understanding detailed metabolism in any species. The red X shows that trout did not have metabolites typically found in humans, the red brackets indicate that the hydroxy-propranolol was not detected but is inferred as that is an intermediate to other metabolites that were found. The green X signifies that a metabolite apparently unique to the dog was not present in trout.

**References**

Hasler J.A., Estabrook R., Murray M., et al., (1999). Human cytochromes P450. *Molecular Aspects of Medicine* 20(1–2), 1-137.

Imamura Y., Wu X., Noda A., Noda H. (2002). Side-chain metabolism of propranolol: involvement of monoamine oxidase and mitochondrial aldehyde dehydrogenase in the metabolism of N-desisopropylpropranolol to naphthoxylactic acid in rat liver. Life Sciences 70(22), 2687-2697.

Masubuchi Y., Hosokawa S., Horie T., Suzuki T., Ohmori S., Kitada M. and Narimatsu S. (1994). Cytochrome P450 isozymes involved in propranolol metabolism in human liver microsomes. The role of CYP2D6 as ring-hydroxylase and CYP1A2 as N-desisopropylase. *Drug Metabolism and Disposition* 22(6), 909-915.

Narimatsu S., Kobayashi N., Masubuchi Y. et al., (2000). Species difference in enantioselectivity for the oxidation of propranolol by cytochrome P450 2D enzymes. *Chemico-Biological Interactions* 127(1), 73-90

Sowinski K.M., Lima J.J., Burlew B.S., Massie J.D. and Johnson J.A. (1996). Racial differences in propranolol enantiomer kinetics following simultaneous i.v. and oral administration. *British Journal of Clinical Pharmacolog*y 42, 339-346.

Walle T., Walle U.K., and Olanoff L.S. (1985). Quantitative account of propranolol metabolism in urine of normal man. *Drug Metabolism and Disposition* 13(2), 204-209.

Wu X., Noda A., Noda H., and Imamura Y. (2001). Side-chain metabolism of propranolol: involvement of monoamine oxidase and aldehyde reductase in the metabolism of N-desisopropylpropranolol to propranolol glycol in rat liver. *Comparative Biochemistry and Physiology Part C: Toxicology & Pharmacology* 129(4), 361-368
